# Intravenous administration of CpG7909 lipoplex enhances anti-PD1 immunotherapy by modulating the tumor microenvironment and inducing durable tumor regression

**DOI:** 10.1038/s41598-025-29622-x

**Published:** 2025-11-25

**Authors:** Chia-Mu Tu, Meng-Hsuan Lin, Chih-Peng Liu, Shih-Ta Chen, Chun-Min Liu, Meng-Ping She, Yu-Cheng Wang, Hsiang-Ching Wang, Yuan-Chia Chang, Li-Wen Chang, Yo-Wen Lo, Min-Xun Lin, Ping-Fu Cheng, Felice Cheng, Jen-Kun Chen, Jun-Lun Meng, Ming-Hsi Wu, Yu-Cheng Su

**Affiliations:** 1 Industrial Development Graduate Program of CEB, National Yang Ming Chiao Tung University, Hsinchu, Taiwan; 2 Biomedical Technology and Device Research Laboratories, Industrial Technology Research Institute, Hsinchu, Taiwan; 3 Institute of Molecular Medicine and Bioengineering, National Yang Ming Chiao Tung University, Hsinchu, Taiwan; 4 Institute of Biomedical Engineering and Nanomedicine, National Health Research Institutes, Miaoli, Taiwan; 5 Department of Biological Science and Technology, Center for Intelligent Drug Systems and Smart Bio-devices (IDS2B), National Yang Ming Chiao Tung University, Room 720, BioICT Building, 75 Bo-Ai Street, Hsinchu, Taiwan; 6 Department of Biomedical Science and Environmental Biology, Drug Development and Value Creation Research Center, Kaohsiung Medical University, Kaohsiung, Taiwan

**Keywords:** Nanobiotechnology, Cancer immunotherapy, Drug discovery

## Abstract

The use of immune checkpoint inhibitors (ICIs) is a potential immunotherapy strategy to prevent immune escape in cancer cells. However, low immune infiltration and low antigen presentation in the tumor microenvironment (TME) have led to ineffective ICI immunotherapy in clinical studies. Although intratumoral injection of Toll-like receptor 9 (TLR9) agonists was shown to improve the efficacy of ICIs by increasing the number of tumor-infiltrating lymphocytes (TILs), it has limited effects on metastatic or deep-seated tumors. To overcome this challenge, we developed CpG7909 (a TLR9 agonist) lipoplex using a cationic lipid formulation, which exhibited preferential accumulation in tumors and spleen following systemic administration, Combination immunotherapy with an anti-PD1 antibody and CpG7909 lipoplex significantly promoted the infiltration of CD8^+^ T cells (> twofold) into the TME of the CT26 tumor animal model and effectively inhibited both deep-seated colorectal and metastatic lung tumors. Furthermore, the complete response (CR) rate of mice receiving concurrent therapy increased from 0 to 75% compared with that of those receiving anti-PD1 monotherapy. To our knowledge, this study is among the first to evaluate the combination of CpG7909 lipoplexes with anti-PD1 antibodies in the context of cancer immunotherapy. The findings suggest that this approach may convert the TME from an immunologically “cold” to “hot” state, thereby enhancing tumor suppression and potentially improving the response rate to ICIs.

**Supplementary Information:**

The online version contains supplementary material available at 10.1038/s41598-025-29622-x.

## Introduction

ICIs have revolutionized cancer therapy by enhancing immune responses to anticancer therapy^1–3^. ICIs can block immune checkpoints on the surface of T-lymphocytes and antigen-presenting cells to restore the anti-tumor activity of CD8^+^ T cells^4,5^. In 2011, Ipilimumab, a CTLA-4 inhibitor, gained FDA approval and became the first ICI drug^6,7^. Another seven ICIs have subsequently gained approval, including PD-1 inhibitors (nivolumab, pembrolizumab, cemiplimab)^8,9^, PD-L1 inhibitors (atezolizumab, durvalumab)^10–12^, and the LAG-3 inhibitor (relatlimab)^13^. PD-1/PD-L1 inhibitors are the most common ICI drugs and have been clinically applied to more than 20 different types of cancer^14,15^. For example, the overall response rate of NSCLC patients (with PD-L1 Tumor Proportion Score (TPS) ≥ 50%, defined as ≥ 50% of tumor cells showing PD-L1 positivity by IHC) to pembrolizumab is 44.8%, whereas that to chemotherapy is 27.8%^16^. Furthermore, pembrolizumab increased progression-free survival to 16.5 months compared with 8.2 months under standard care in patients with metastatic colorectal cancer diagnosed as microsatellite instability-high (MSI-H)^17^.

Although ICIs enhance clinical benefits with durable responses, the response rate remains low in most cancers (10–35%)^18,19^. Recent studies have emphasized the importance of the TME in dictating responses to ICIs and dividing tumors into immunogenic (hot) and nonimmunogenic (cold) categories^20–22^. The microsatellite stability type is found in 80‒85% of colorectal cancer patients and is also characterized as a ‘cold’ tumor ^23,24^. The KEYNOTE-164 study revealed almost no response to pembrolizumab in MSS colorectal cancer patients^24^. Similarly, patients with MSS gastric cancer treated with nivolumab in combination with chemotherapy had a significantly lower median overall survival (14.3%) than did patients with MSI-H tumors (44.8%) in the CheckMate-649 study^25^.

Resistance to ICIs is influenced primarily by the TME, particularly the presence and functionality of TILs^26,27^. Fully effective ICIs require preexisting antitumor immunity, particularly the presence of CD8^+^ T cells among TILs in the TME^28–30^. Therefore, a crucial strategy to overcome hypo-responses to ICIs is the integration of multimodality therapy to recruit immune cells into tumor tissue and concurrently alter immune phenotypes from cold to hot^31–33^. Several studies have suggested that TLR9 agonists have potential in combination with ICIs to promote immune activation, converting cold tumors into hot tumors^34–36^. To further improve the stability and efficacy of TLR9 agonists, such as MGN-1703^37–39^ and IMO-2125^40,41^, dumbbell-shaped structural and phosphorothioate modifications were applied^42,43^. Furthermore, CMP-001 and AST-008 are nanoparticle-encapsulated TLR9 agonists that have shown enhanced stability and cellular uptake in preclinical studies and elevated response rates to ICIs in clinical trials. However, owing to safety concerns related to off-target side effects, these TLR9 agonists are limited to intratumoral administration. As a result, their therapeutic effects are often confined to injected tumor lesions, reducing their efficacy against metastatic or deep-seated tumors. In contrast, CpG7909 lipoplex is formed through the electrostatic interaction between cationic lipids and negatively charged CpG7909 oligodeoxynucleotides. Its formulation, featuring specific lipid ratios and polyethylene glycol (PEG)-modified lipids, enables improved stability, prolonged circulation time, and preferential accumulation in immune-related organs such as the spleen and lymph nodes when administered intravenously. These attributes make CpG7909 lipoplex a promising alternative for systemic treatment of cancers and immunological disorders^44–48^.

Importantly, accumulating evidence indicates that the spleen plays a critical role in orchestrating effective antitumor immunity. The spleen, along with bone marrow and lymph nodes, constitutes an interconnected immunological network perturbed in many tumor types^29^. Studies have shown that the spleen acts as a secondary lymphoid organ where robust immune priming and proliferation occur, including the expansion of plasma cells, Th1-biased T cells, and NK cells during tumor rejection^49^. In murine models, the spleen was also demonstrated to facilitate antitumor immune responses against melanoma, indicating its relevance in both experimental and potentially clinical settings^50^. Furthermore, preferential accumulation of immunomodulators in the spleen has been shown to enhance IFN-γ secretion and support durable antitumor immune memory^51^. These findings underscore the rationale for leveraging spleen-accumulating nanomedicine platforms to augment systemic immune activation and overcome the spatial limitations of intratumoral immunotherapy delivery. Given the unmet clinical need for treating metastatic and deep-seated tumors, in this study, we demonstrate that CpG7909 lipoplex preferentially accumulates in the spleen, activates immune cell populations, and in combination with ICIs, significantly enhances TIL infiltration and tumor regression in both subcutaneous and metastatic colorectal cancer models. This work establishes CpG7909 lipoplex as a novel platform for systemic TLR9 activation, capable of converting cold tumors into hot and overcoming current limitations in immunotherapy delivery.

## Methods

### Preparation of CpG7909 lipoplex

CpG7909 (GenePharma Co., Ltd.) was incorporated into cationic lipid nanoparticles prepared via the thin-film hydration method. Cationic lipids (e.g., DOTAP) and helper lipids (e.g., cholesterol and DSPE-PEG) were dissolved in chloroform at a total lipid concentration of 10 mg/mL, evaporated to form a thin lipid film, and hydrated with 10 mM Tris(hydroxymethyl)aminomethane buffer. Nanoparticles were formed by sonication using a Misonix sonicator-XL2020 (Misonix, Farmingdale, NY, USA) at 35% amplitude for 20 minutes at a temperature of 10 °C. During the sonication process, particle size was monitored as an in-process control until it reached the target range of 70–120 nm, after which the lipid nanoparticles were filtered once through a 0.22 μm membrane to remove aggregates and ensure uniform particle size distribution.

The final CpG7909 formulation (CpG7909 lipoplex) was prepared in a 150 mL container equipped with a PTFE cross-type stir bar and operated at 800 rpm. CpG7909 solution was added at an N/P ratio of 4.8 and a feeding rate of 6 mL/min, followed by continuous stirring for 40–60 min to promote complete electrostatic complexation and uniform particle formation, yielding a final product with a particle size below 200 nm.

The resulting CpG7909 lipoplex exhibited an average particle size of 167.6 nm, a PDI of 0.181, and a zeta potential of + 32.8 mV, indicating a uniform and positively charged nanoparticle population. After solid-phase extraction (SPE) to separate unbound CpG7909, the free CpG7909 content was found to be < 1%, indicating an encapsulation efficiency (EE) greater than 99%.

### Cell Lines and reagents

The following cell lines were used in this study: CT26 and EMT6 (both from ATCC), luciferase-labeled CT26 cells (GeneCopoeia), HEK293-hTLR9/NF-κB-luc cells (BPS Bioscience), and RAW-Blue™ mouse macrophage cells (InvivoGen). Female BALB/c mice (6–8 weeks old, ~ 20 g) were purchased from BioLASCO Taiwan. F-CpG7909 and FAM-F-CpG7909 were obtained from MDBio and Integrated DNA Technologies (IDT), respectively.

### TLR9 activity assay

TLR9 activation was assessed using HEK293-hTLR9/NF-κB-luc cells. Cells were seeded at a density of 1 × 10^4^ cells per well in 96-well plates and treated with CpG7909 lipoplex or F-CpG7909 at concentrations ranging from 8 to 4000 nM. After 6-h incubation at 37 °C in a 5% CO_2_ atmosphere, the supernatants were collected, and the activity of secreted alkaline phosphatase was measured using the Quanti-Blue assay (InvivoGen) as an indicator of NF-κB activation. Dose–response curves were generated, and EC_50_ values were calculated to compare activation efficiencies.

### In vitro cellular uptake assay

RAW-Blue™ mouse macrophage cells were plated at a density of 1 × 10^5^ cells per well onto sterile coverslips that had been placed in 24-well plates and incubated overnight. Cells were treated with 100 nM FAM-F-CpG7909 or FAM-CpG7909 lipoplex for 1 h at 37 °C. After incubation, cells were washed three times with phosphate-buffered saline (PBS), fixed with 4% paraformaldehyde for 15 min, and the coverslips were mounted onto glass slides using mounting medium. Intracellular uptake was visualized using confocal laser scanning microscopy, and fluorescence signals were quantified using ImageJ software. Fluorescence intensity was analyzed to evaluate the efficiency of cellular uptake.

### PBMC isolation and stimulation

PBMCs were isolated from healthy donors using Ficoll-Paque PLUS density gradient centrifugation. PBMCs were seeded at a density of 1 × 10^6^ cells per well in 96-well plates and stimulated with F-CpG7909 (100 nM) or CpG7909 lipoplex (10–100 nM) for 24 h at 37 °C in RPMI-1640 medium supplemented with 10% fetal bovine serum. Cytokine concentrations in the supernatants were measured using a multiplex immunoassay.

### Tumor growth and treatment evaluation in CT26 syngeneic models

Cultured CT26 cells were harvested by trypsinization, washed twice with PBS, and resuspened in PBS at 2 × 10^6^ cells/mL. A total of 2 × 10^5^ cells (in 100 µL PBS) were injected subcutaneously into the right flank of each BALB/c mouse to establish tumors. Tumor volumes were monitored, and once they reached 75–200 mm^3^, the mice were randomized into treatment groups (*n* = 8 per group). Treatment groups included vehicle control (intraperitoneal [ip] injection), anti-PD1 antibody (250 µg/mouse, ip, twice weekly), F-CpG7909 (15 µg/mouse, intravenous [iv] injection, once weekly) combined with anti-PD1 antibody (250 µg/mouse, ip, twice weekly), or CpG7909 lipoplex (1.5–15 µg/mouse, iv, once weekly) combined with anti-PD1 antibody (250 µg/mouse, ip, twice weekly). Tumor volumes were measured at regular intervals to evaluate treatment efficacy.

### Ex vivo Immunohistochemistry and H&E Staining

The BALB/c mice were subcutaneously injected with CT26 cell suspension (2 × 10^5^ cells in PBS). After tumor inoculation (confirmed to have grown to 75–200 mm^3^ within 10 days), the mice were randomly assigned into four groups (*n* = 5–6 mice per group): vehicle control, anti-PD1 antibody (250 µg/mouse, ip injection twice a week), CpG7909 lipoplex (15 µg/mouse, iv injection once a week) combined with anti-PD1 antibody (250 µg/mouse, ip twice a week), and F-CpG7909 (15 µg/mouse, iv once a week) combined with anti-PD1 antibody (250 µg/mouse, ip twice a week). Approximately 2–3 weeks after the treatment initiation, the mice were euthanized using CO_2_, and their tumors were excised. Tumor tissues were fixed in 10% neutral buffered formalin for at least 24 h, followed by paraffin embedding and sectioning. Immunohistochemical staining was carried out on 5-µm tumor sections using a rabbit monoclonal anti-mouse CD8 alpha antibody (EPR20305, Abcam). CD8^+^ T-cell infiltration was visualized under a microscope imaging system (Olympus BX51), and the number of CD8^+^ cells was quantified in randomly selected fields. The results were statistically analyzed to evaluate differences in TIL recruitment among treatment groups.

### Re-challenge experiment for long-term immunity assessment

BALB/c mice that achieved complete tumor regression following combination treatment with CpG7909 lipoplex (15 µg/mouse, iv, once weekly) and anti-PD1 antibody (250 µg/mouse, ip, twice weekly) were selected for re-challenge studies. To evaluate immune memory, these mice were subcutaneously re-injected with the same CT26 tumor cells (2 × 10^5^). After confirming that the mice were resistant to CT26 tumor re-establishment, a subset of the mice was further challenged with a different tumor type, EMT6 breast cancer cells (2 × 10^5^), to assess the tumor specificity of the immune memory response.

### Assessment of tumor-specific T-cell responses using IFN-γ ELISPOT assays

Splenocytes were harvested from BALB/c mice treated with CT26 tumors until tumor regression was achieved, or from naïve BALB/c mice. Spleens were aseptically removed, gently homogenized through a 70-µm cell strainer into cold RPMI-1640 medium, followed by erythrocyte lysis using ACK lysis buffer (Lonza). After washing twice with PBS, single-cell suspensions were counted and used for downstream assays. Tumor-specific T-cell responses were assessed using IFN-γ ELISPOT assays. ELISPOT plates (Mabtech, Cincinnati, OH) were pre-coated with an anti-IFN-γ antibody (eBioscience), and 1 × 10^6^ mouse splenocytes were added per well. Splenocytes were incubated either with 10 μg/mL of AH1 peptide (AnaSpec, CA) to stimulate tumor-specific T cells, or with a stimulation cocktail containing 1 μM PMA and 10 ng/mL Ionomycin (eBioscience) as a positive control. Plates were incubated for 24 h, followed by the addition of IFN-γ detection antibodies. IFN-γ spots were visualized and quantified using an ELISPOT assay kit (Mabtech, Cincinnati, OH).

### Lung metastasis model

To model lung metastasis, BALB/c mice were injected intravenously with 2.5 × 10^5^ CT26-luciferase cells. On day 5 post-injection, mice were randomized into treatment groups (*n* = 10 per group): vehicle, anti-PD1 antibody, CpG7909 lipoplex plus anti-PD1, F-CpG7909 plus anti-PD1, or oxaliplatin (positive control). Tumor burden was measured weekly using bioluminescence imaging with an IVIS system.

### Pharmacokinetics and Biodistribution

CpG7909 lipoplex and F-CpG7909 labeled with [^125^I] were administered intravenously to tumor-bearing mice. Blood and tissues (e.g., liver, spleen, kidneys, and tumors) were collected at predetermined time points. Radioactivity was measured using a gamma counter. Pharmacokinetic parameters, such as half-life and clearance, were calculated using Phoenix WinNonlin software.

### Safety evaluation

BALB/c mice received vehicle or CpG7909 lipoplex (15 μg) intravenously once weekly for two weeks. Mice were monitored for changes in body weight, clinical signs of toxicity, and serum liver enzyme levels [Alanine aminotransferase (ALT) and Aspartate aminotransferase (AST)] to evaluate systemic safety.

### Statistical analysis

Data were expressed as mean ± standard deviation (SD). Statistical significance between two groups was determined using a two-tailed unpaired Student’s t-test, whereas comparisons among multiple groups were analyzed using the Kruskal–Wallis test followed by Dunn’s multiple comparisons test. A *p* value < 0.05 was considered statistically significant.

## Results

### CpG7909 lipoplex enhances TLR9 activation by increasing cellular uptake

**Fig. 1:**
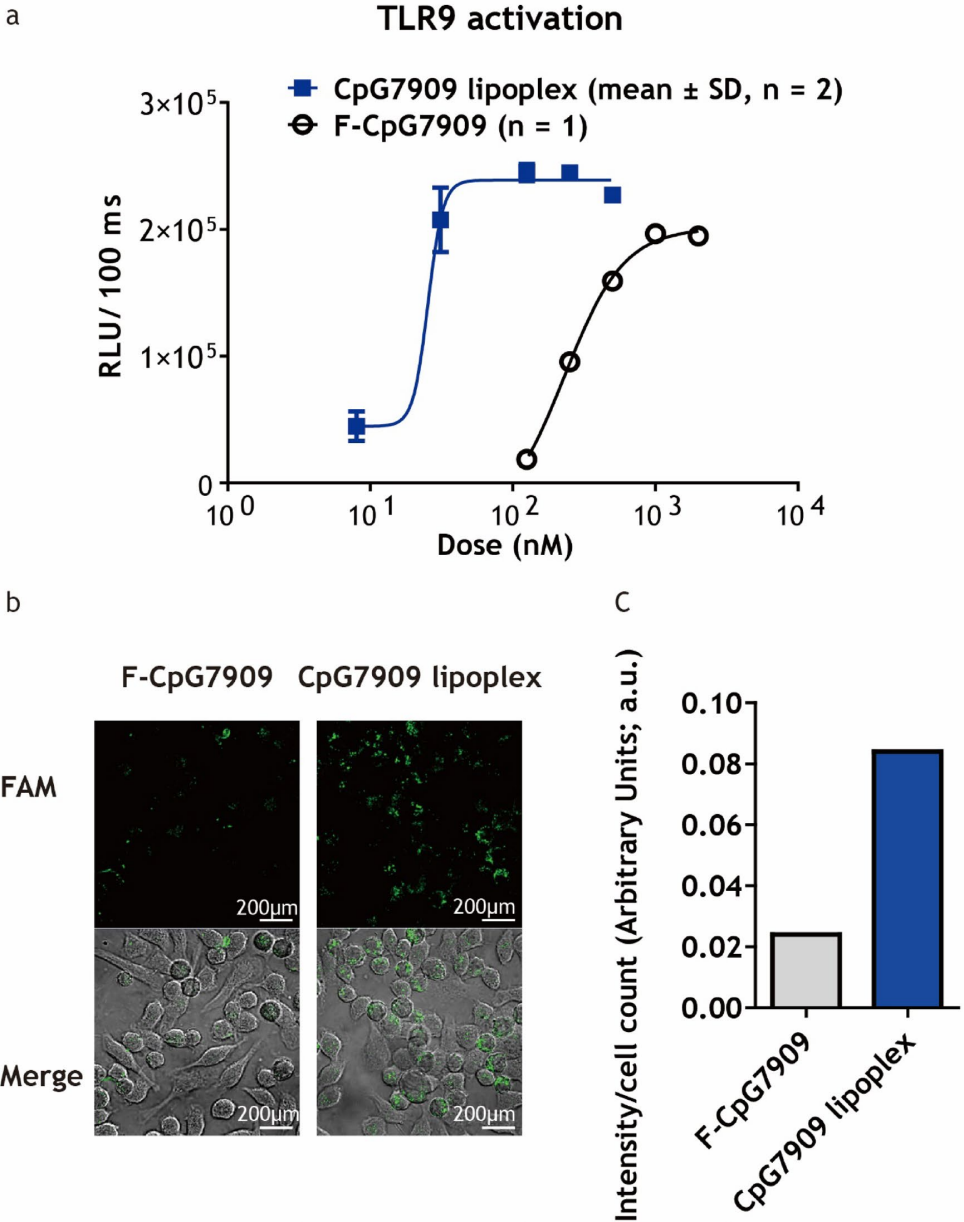
TLR9 Activation and Cellular Uptake of CpG7909 Lipoplex. (**a**) HEK293-hTLR9/NF-κB-luc cells were treated with CpG7909 lipoplex or free CpG7909 (F-CpG7909). Data for CpG7909 lipoplex are shown as mean ± SD (*n* = 2); F-CpG7909 was tested once (*n* = 1). RLU/100 ms represents luciferase activity indicating TLR9 activation. The calculated EC_50_ values were 25.4 nM for CpG7909 lipoplex and 229.6 nM for F-CpG7909, respectively. (**b**) Cellular uptake of F-CpG7909 and CpG7909 lipoplex in RAW-Blue™ cells. Both formulations were labeled with 6-FAM and visualized by confocal microscopy after a 1-h incubation. Merged images show fluorescence (green) overlaid with cell morphology, confirming intracellular localization. Scale bar = 200 μm. (**c**) Quantitative analysis of the fluorescence intensity shown in panel B, normalized to cell count. CpG7909 lipoplex exhibited higher intracellular fluorescence intensity, indicating greater cellular uptake compared with free CpG7909.

HEK293-hTLR9/NF-κB- (Nuclear Factor kappa-light-chain-enhancer of activated B cells) luc cells were used to evaluate the enhancement of TLR9 activation by the CpG7909 lipoplex. The half-maximal effective concentration (EC_50_) for TLR9 activation was measured via a luciferase assay (Fig. 1a). The CpG7909 lipoplex showed a lower EC_50_ (25.4 nM) compared to Free-CpG7909 (F-CpG7909, 229.6 nM), indicating more efficient TLR9 activation. To explore the underlying mechanism, the mouse macrophage cell line RAW-Blue™ was used to measure cellular uptake via confocal microscopy. After 1 h of incubation, the intracellular fluorescence signal was significantly stronger in cells treated with the CpG7909 lipoplex compared with those treated with F-CpG7909 (Fig. 1b), suggesting enhanced cellular uptake of the lipoplex formulation. Quantitative analysis of fluorescence intensity normalized to cell count (Fig. 1c) demonstrated approximately a four-fold increase in signal for CpG7909 lipoplex compared with F-CpG7909, confirming the enhanced cellular uptake efficiency of the lipoplex formulation.

### CpG7909 lipoplex induces robust pro-inflammatory cytokine release from human peripheral blood mononuclear cells (PBMCs)

**Fig. 2:**
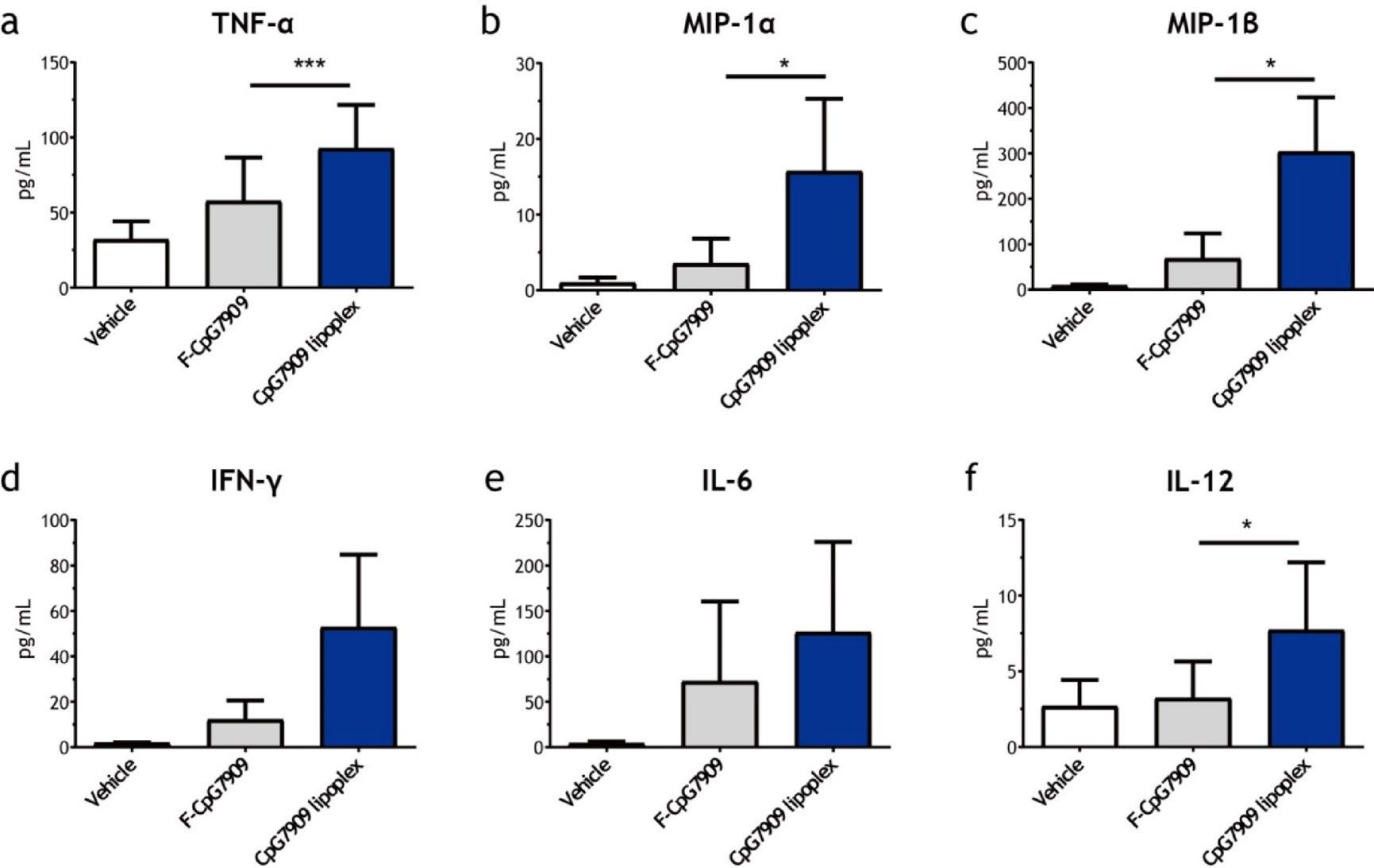
Cytokine Release Induced by CpG7909 Lipoplex in Human PBMCs. Cytokine production by human peripheral blood mononuclear cells (PBMCs; *n* = 3) stimulated with F-CpG7909 or CpG7909 lipoplex for 24 h. Cytokine levels were measured using the Bio-Plex™ Pro assay. Data shown: (**a**) TNF-α, (**b**) MIP-1α, (**c**) MIP-1β, (**d**) IFN-γ, (**e**) IL-6, and (**f**) IL-12. Statistical significance: *p < 0.05, **p < 0.01, ***p < 0.001. Data are presented as mean ± standard error.

Human PBMCs were incubated with 100 nM F-CpG7909 or CpG7909 lipoplex, and the levels of the resulting proinflammatory cytokines and chemokines were quantified. Compared with F-CpG7909, CpG7909 lipoplex treatment led to significant increases in TNF-α, MIP-1α, MIP-1β, and IL-12 production (Fig. 2a,b,c, and f). Notably, TNF-α levels were markedly elevated (*p* < 0.001), whereas MIP-1α, MIP-1β, and IL-12 levels were significantly increased (*p* < 0.05). Although IFN-γ levels were elevated in the CpG7909 lipoplex-treated group, the increase did not reach statistical significance (*p* = 0.056) (Fig. 2d), indicating a trend toward increased immune activation. Interestingly, there was no significant increase in IL-6 production (Fig. 2e), suggesting that the CpG7909 lipoplex can stimulate immune responses without excessive inflammatory side effects.

### Intravenous CpG7909 lipoplex and anti-PD1 combination therapy enhances antitumor efficacy and promotes complete responses in CT26 tumor model

**Fig. 3:**
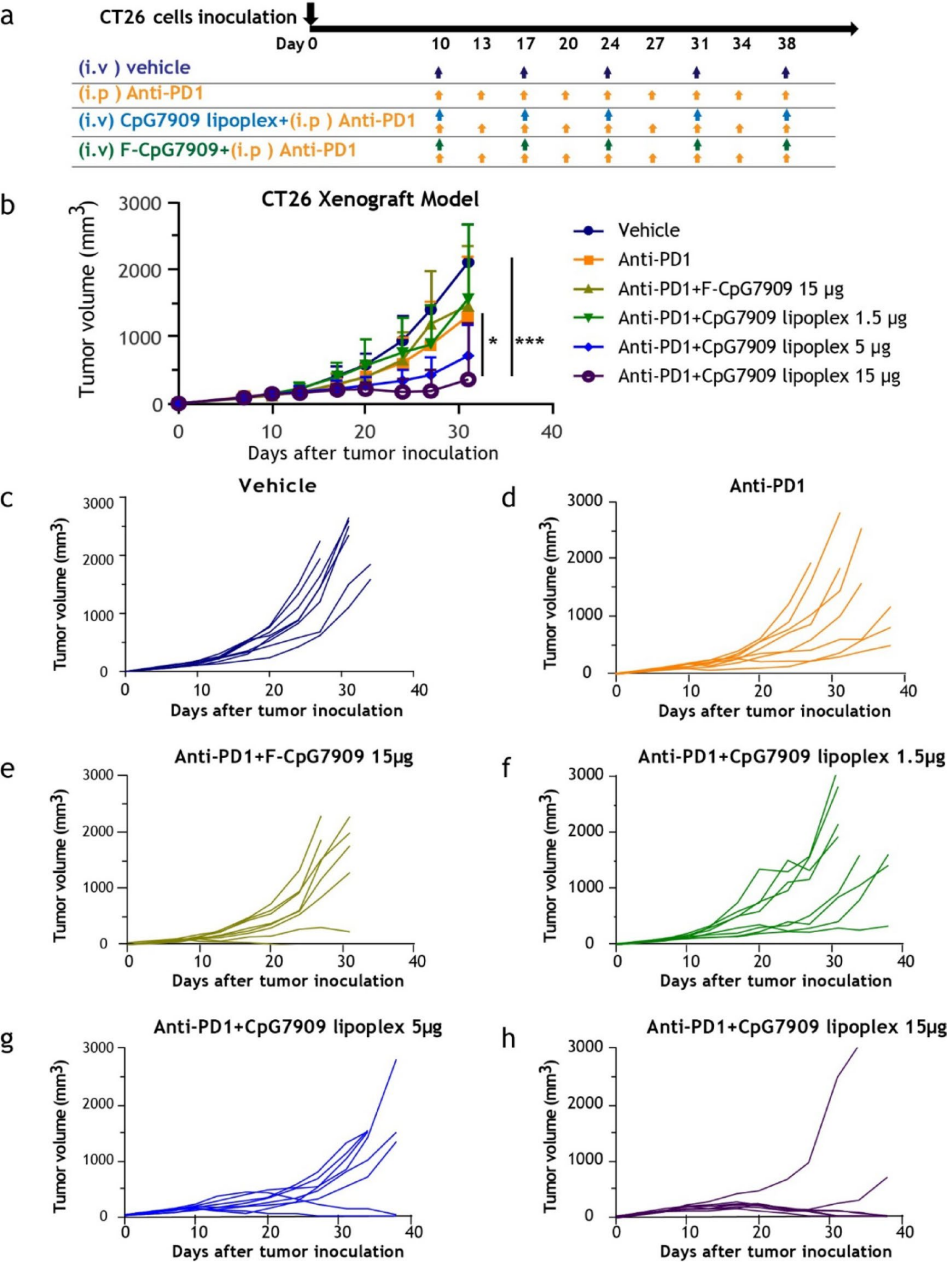
Antitumor Activity of CpG7909 Lipoplex and Anti-PD1 Therapy in CT26 Tumor Model. (**a**) Treatment schedule for CT26 syngeneic colorectal cancer model using vehicle, anti-PD1 antibody (monotherapy), or anti-PD1 combined with CpG7909 lipoplex (1.5, 5, or 15 μg/mouse). (**b**) Tumor growth curves for each treatment group. (c–h) Individual tumor growth plots: (**c**) vehicle, (**d**) anti-PD1, (**e**) anti-PD1 + F-CpG7909 (15 μg), (**f**) anti-PD1 + CpG7909 lipoplex (1.5 μg), (**g**) 5 μg, and (**h**) 15 μg. Animals exceeding a tumor volume of 1688 mm^3^ or > 20% body weight loss were euthanized according to ethical guidelines. Tumor volumes are shown as mean ± standard error. Statistical significance: *p < 0.05, ***p < 0.001.

The antitumor efficacy of intravenous CpG7909 lipoplex combined with anti-PD1 antibody was evaluated in a subcutaneous CT26 tumor model. Mice received CpG7909 lipoplex at doses of 1.5, 5, or 15 μg/mouse, each in combination with anti-PD1 antibody (Fig. 3a). The CT26 murine colon carcinoma model was chosen for this study due to its well-characterized immunogenic properties and its ability to mimic key features of human colorectal cancer, making it a widely used model for evaluating immunotherapies. The combination therapy, particularly at the highest dose of CpG7909 lipoplex (15 μg/mouse), inhibited tumor growth, achieving a tumor growth inhibition (TGI) rate of 89% on day 31 compared with 41% in the anti-PD1 antibody alone group (Fig. 3b). Dose-dependent antitumor effects were observed, with TGI values of 28%, 71%, and 85% for CpG7909 lipoplex doses of 1.5, 5, and 15 μg/mouse, respectively. Notably, 75% of mice (6/8) treated with 15 μg/mouse CpG7909 lipoplex plus anti-PD1 antibody achieved complete responses (CRs), while no CRs were observed in the anti-PD1 monotherapy group (Fig. 3c–h). Individual tumor growth curves further confirmed that CpG7909 lipoplex treatment resulted in durable tumor regression in a subset of animals at the higher dose (Fig. 3h).

**Fig. 4:**
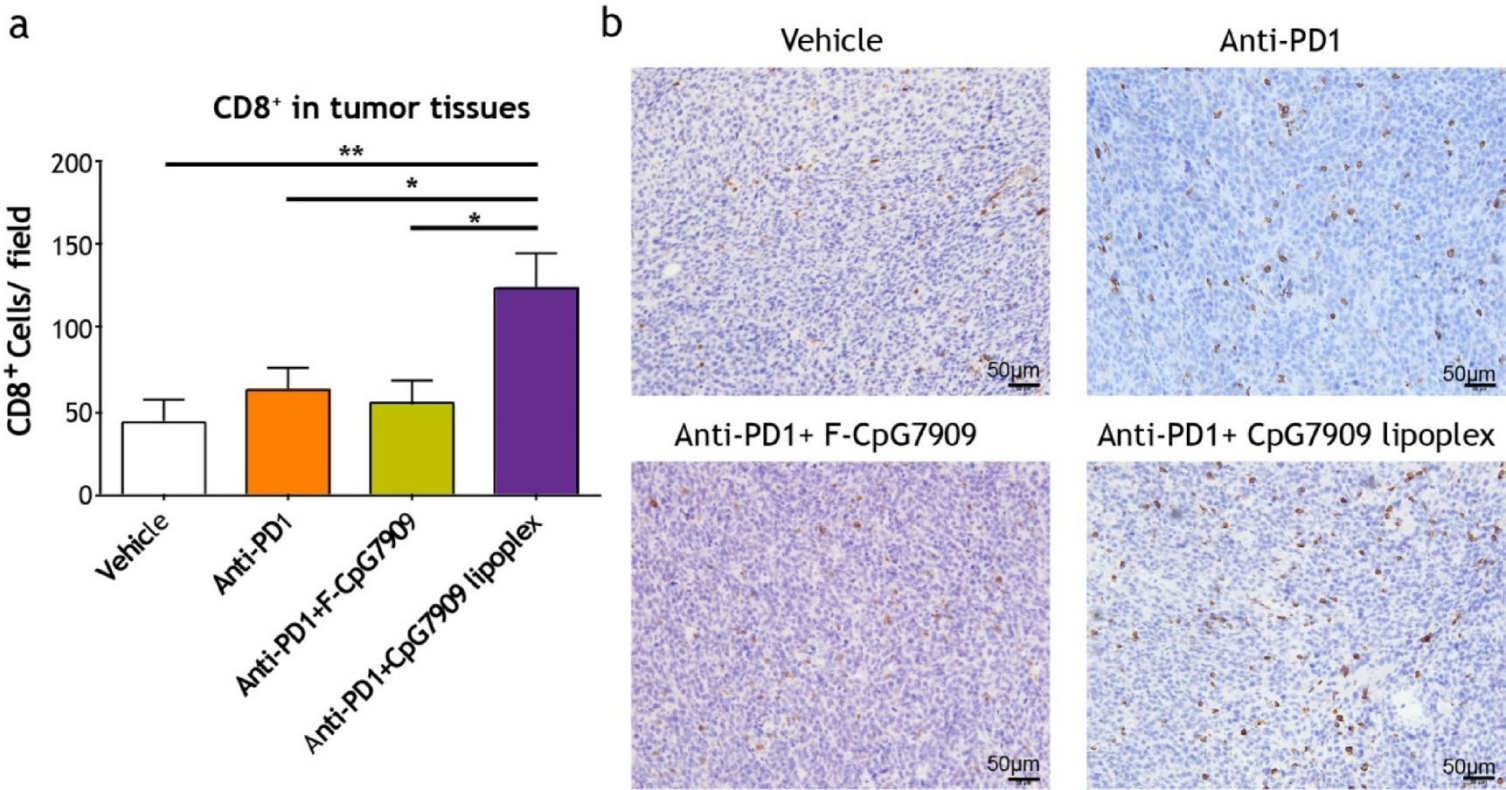
Increased CD8^+^ Tumor-Infiltrating Lymphocytes Induced by CpG7909 Lipoplex and Anti-PD1 Therapy. (**a**) Quantification of CD8^+^ T cells in CT26 tumor tissues from mice treated with vehicle, anti-PD1, anti-PD1 + F-CpG7909, or anti-PD1 + CpG7909 lipoplex. Statistical significance: *p < 0.05, **p < 0.01. Data are presented as mean ± standard error (*n* = 5–6). (**b**) Representative IHC images of tumor sections stained for CD8^+^ T cells. Images correspond to vehicle, anti-PD1, anti-PD1 + F-CpG7909, and anti-PD1 + CpG7909 lipoplex treatment groups. Scale bar = 50 μm.

Immunohistochemistry analysis revealed a significant increase in CD8^+^ T-cell infiltration within the tumors of the mice treated with the combination of the CpG7909 lipoplex and anti-PD1 antibody (Fig. 4a,b). Quantification and statistical analysis confirmed that this combination therapy significantly increased the number of CD8^+^ TILs compared with the vehicle group (*p* < 0.01), the anti-PD1 + F-CpG7909 group (*p* < 0.05), and the anti-PD1 monotherapy group (*p* < 0.05). These findings support the hypothesis that increased TIL recruitment contributes to the antitumor response.

### CpG7909 lipoplex induces tumor-specific immune memory

**Fig. 5:**
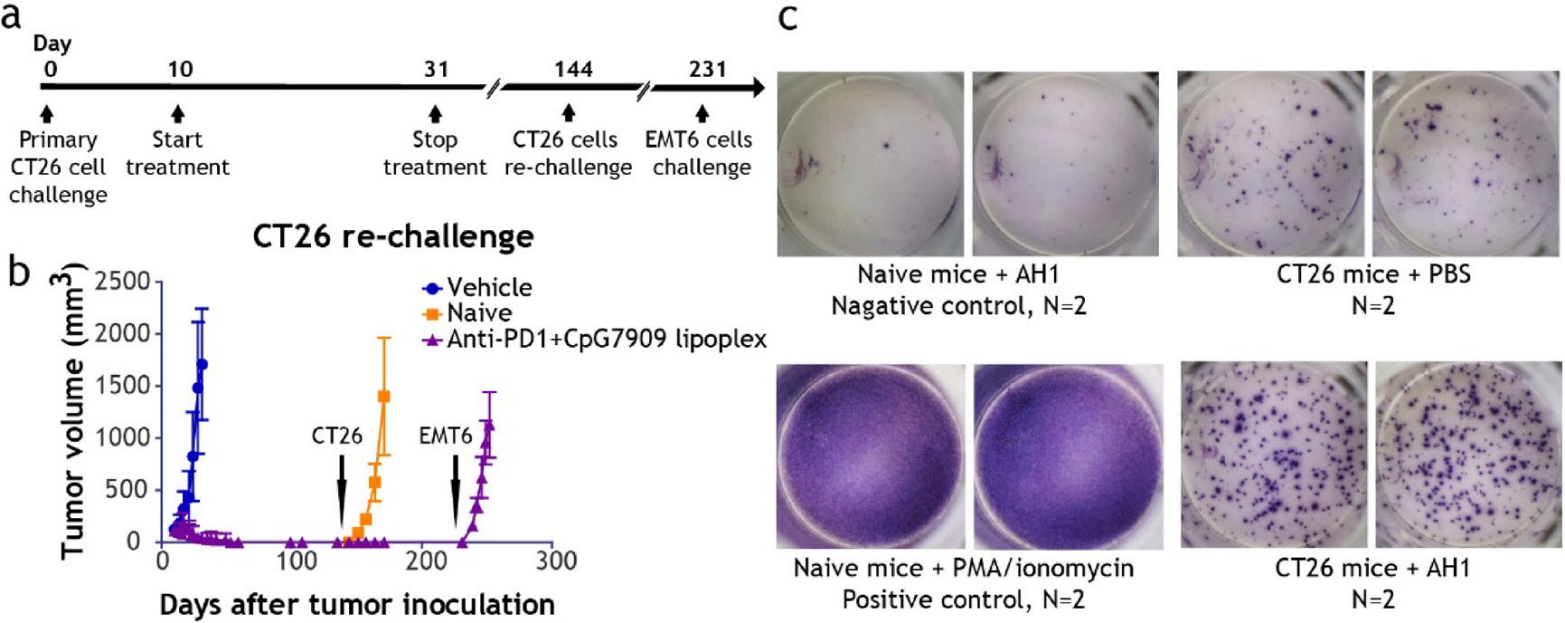
CpG7909 Lipoplex Promotes Long-term Tumor Immunity and Antigen-Specific T-Cell Responses. (**a**) Schematic of the experimental timeline for primary CT26 tumor inoculation, treatment, and tumor re-challenges in BALB/c mice. (**b**) Tumor volume progression during CT26 tumor re-challenge in vehicle-treated, naïve, and CpG7909 lipoplex + anti-PD1-treated mice. Mice cured by combination therapy showed complete protection against rechallenge, indicating durable tumor-specific immunity. (**c**) IFN-γ ELISPOT assay using splenocytes from cured and control mice. Cells were stimulated with AH1 peptide (tumor-specific antigen) or PMA/ionomycin (positive control). Representative wells demonstrate enhanced antigen-specific T-cell responses in CpG7909 lipoplex-treated mice.

To evaluate the establishment of long-term antitumor memory, a tumor rechallenge study was conducted (Fig. 5a). Mice that achieved a CR following treatment with CpG7909 lipoplex and anti-PD1 antibody combination therapy were rechallenged with CT26 cells on day 144. As shown in Fig. 5b, mice previously cured with CpG7909 lipoplex and anti-PD1 therapy effectively rejected the CT26 tumor rechallenge, with no detectable tumor growth. In contrast, both vehicle-treated and naïve mice developed tumors, indicating the induction of robust tumor-specific immune memory. However, these mice failed to reject a subsequent challenge with EMT6 breast cancer cells on day 231, indicating that the immune memory was specific to CT26-derived antigens. Furthermore, IFN-γ ELISPOT assays (Fig. 5c) using splenocytes from cured mice showed a significant increase in IFN-γ secretion upon stimulation with the CT26-associated AH1 antigen. These results further confirm the induction of tumor-specific immune memory by the CpG7909 lipoplex and anti-PD1 combination therapy.

### Intravenous CpG7909 lipoplex inhibits lung metastasis in colorectal cancer

**Fig. 6:**
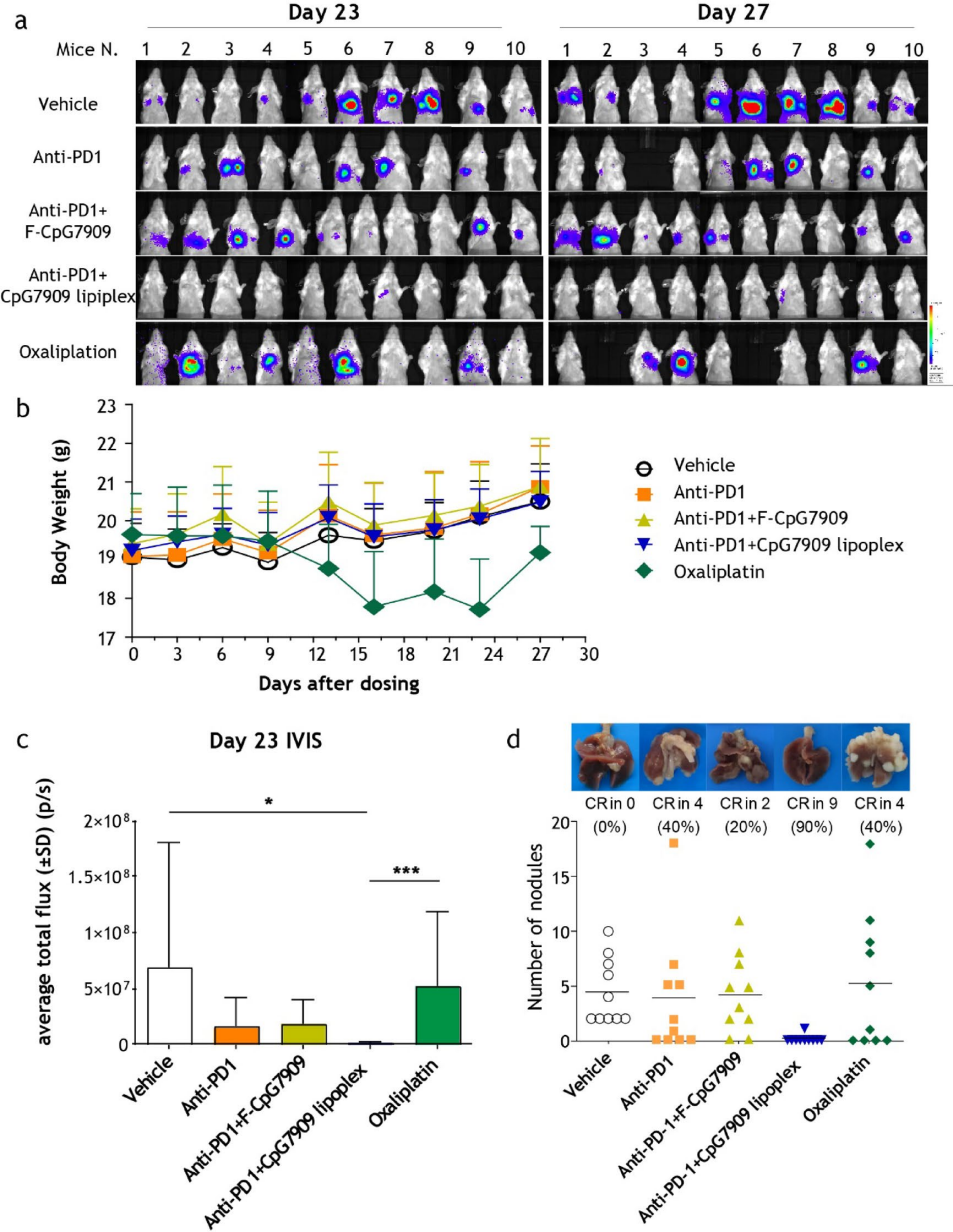
IVIS Imaging and Lung Metastasis Assessment of CpG7909 Lipoplex and Anti-PD1 Combination Therapy. (**a**) Representative IVIS images of CT26-luciferase lung metastasis-bearing mice on days 23 and 27 post-inoculation, following treatment with vehicle, anti-PD1, anti-PD1 + CpG7909 lipoplex, or oxaliplatin. (**b**) Body weight changes over the course of treatment. (**c**) Quantification of luminescent signal intensity on day 23. Statistical analysis was performed using the Kruskal–Wallis test followed by Dunn’s multiple comparisons test. *p* < 0.05 (*), *p* < 0.001 (***). (**d**) Number of lung metastatic nodules in each treatment group. Data are presented as means ± standard error.

In a lung metastasis model using CT26-luc cells, the combination of CpG7909 lipoplex and anti-PD1 antibody significantly reduced tumor burden, as assessed by bioluminescence imaging (Fig. 6a). Mice treated with the CpG7909 lipoplex plus anti-PD1 antibody showed a significantly greater reduction in tumor burden compared to the control, F-CpG7909, anti-PD1, and oxaliplatin groups (Fig. 6a). Oxaliplatin, an FDA-approved chemotherapy for colorectal cancer, was used as the positive control in this study to represent the current standard of care. Tumor burden, was quantified based on total bioluminescent flux, was significantly reduced in the Anti-PD1 + CpG7909 lipoplex group compared with the Oxaliplatin (*p* < 0.001) and Vehicle (*p* < 0.05) groups. Although the differences relative to the Anti-PD1 or Anti-PD1 + free CpG7909 groups did not reach statistical significance, the lipoplex-treated group demonstrated a clear trend toward improved tumor control (Fig. 6c). Furthermore, this combination treatment achieved a 90% CR rate based on lung nodule counts (Fig. 6b), which was significantly higher than the CR rates observed in the oxaliplatin group (40%) and the anti-PD1 antibody group (0%). Body weight analysis (Fig. 6d) revealed that while oxaliplatin treatment exhibited partial tumor-inhibitory effects, it caused noticeable fluctuations in body weight, indicating potential treatment-related toxicity. In contrast, mice treated with the CpG7909 lipoplex combination therapy maintained stable body weights throughout the study, highlighting its superior safety and tolerability compared to oxaliplatin.

### CpG7909 lipoplex exhibits spleen-accumulating properties in its pharmacokinetic and tissue distribution profiles

**Fig. 7:**
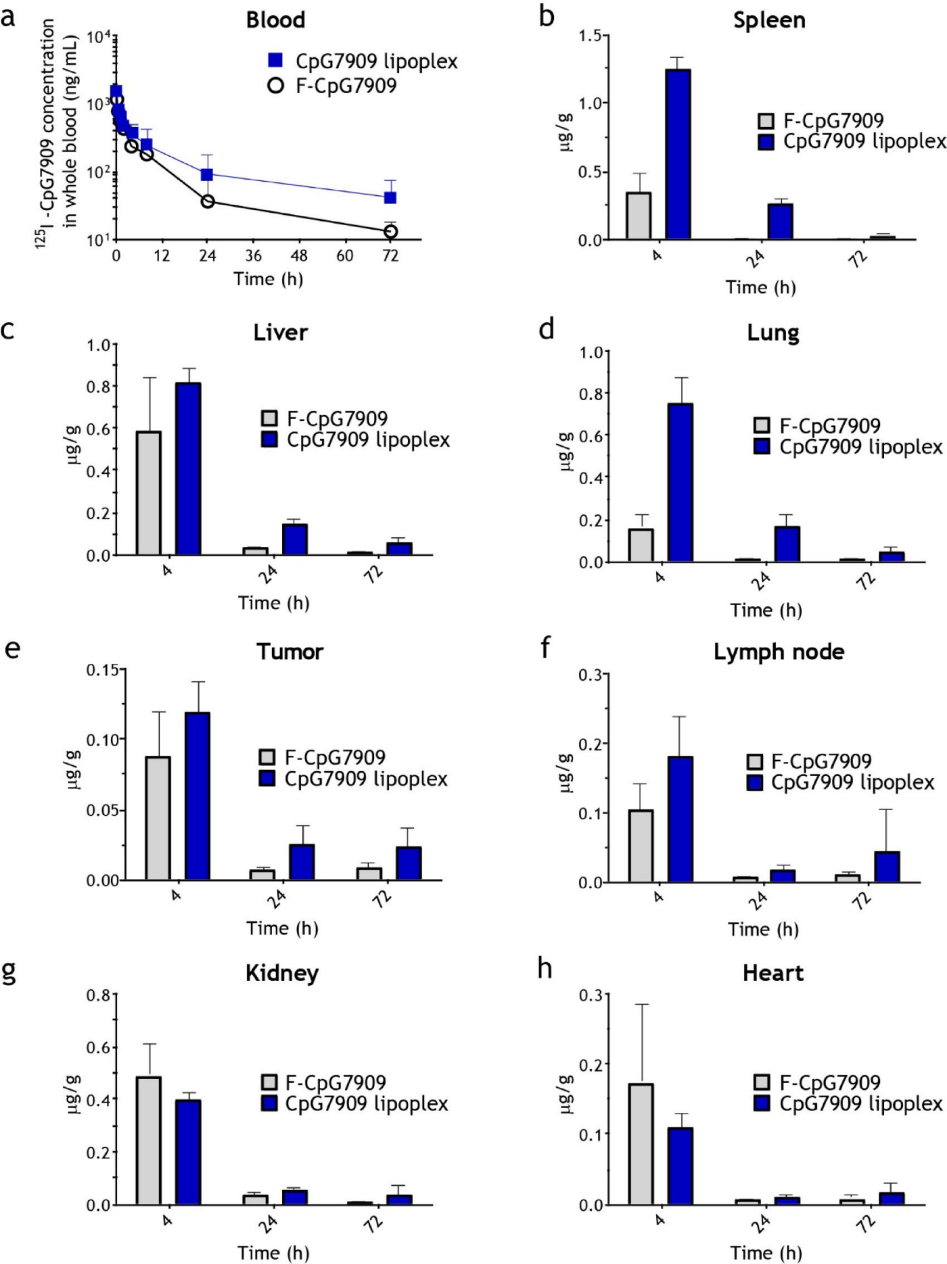
Pharmacokinetics and Tissue Distribution of CpG7909 Lipoplex. (**a**) Concentration–time curve of CpG7909 lipoplex and F-CpG7909 in whole blood following intravenous administration (15 μg/mouse). (B–H) Tissue distribution of CpG7909 lipoplex and F-CpG7909 in (**b**) spleen, (**c**) liver, (**d**) lung, (**e**) tumor, (**f**) lymph node, (**g**) kidney, and (**h**) heart at 1 and 72 h post-administration. Data are presented as means ± standard error, *n* = 3 per group.

Pharmacokinetic analysis (Fig. 7a) revealed that CpG7909 lipoplex exhibited slower clearance and a higher area under the curve (AUC) compared to F-CpG7909, consistent with the extended circulation profile typically observed in liposomal formulations (Supplementary Table S1). In terms of biodistribution, CpG7909 lipoplex showed significantly increased accumulation in various organs, including the spleen, liver, lungs, tumors, and lymph nodes, compared to F-CpG7909 (Fig. 7b–h). The accumulation levels ranged from 3 to 24-fold higher, depending on the tissue and time point assessed (4, 24, or 72 h). This suggests that CpG7909 lipoplex maintains a prolonged circulation time and extended tissue retention, with its enhanced distribution persisting up to 72 h post-administration. Notably, the spleen demonstrated the most significant increase in drug concentration, reaching a 27-fold increase at 24 h, indicating preferential accumulation of the CpG7909 lipoplex in this organ. In contrast, no significant differences were observed in the drug distribution to the kidneys or heart between the CpG7909 lipoplex and F-CpG7909 groups, indicating comparable safety profiles in these organs.

### CpG7909 lipoplex demonstrates favorable tolerability and safety following intravenous administration in mice

**Fig. 8:**
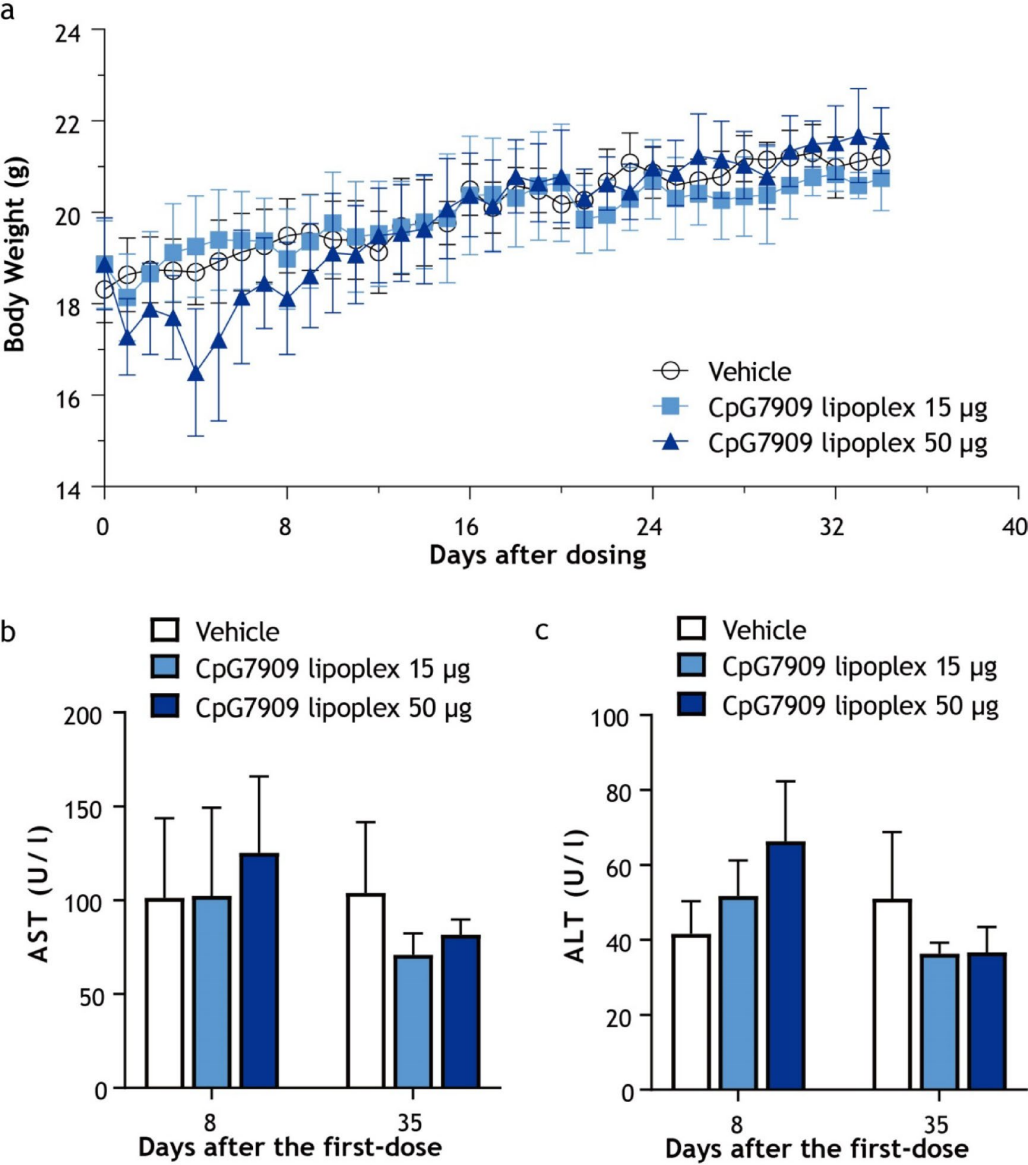
Impact of CpG7909 Lipoplex on body weight and liver function in mice. (**a**) Body weight measurements over 40 days for mice treated with vehicle, CpG7909 lipoplex (15 μg), or CpG7909 lipoplex (50 μg). (**b**) Serum AST levels on days 8 and 35 following treatment. (**c**) Serum ALT levels on days 8 and 35 following treatment. Data are presented as means ± standard error (*n* = 3 per group).

This study demonstrated that BALB/c mice tolerated two weekly intravenous doses of CpG7909 lipoplex (15 or 50 μg/mouse) without significant adverse effects. Body weights remained stable throughout the treatment period in both CpG7909 lipoplex groups, with no significant differences compared to vehicle-treated controls, supporting the safety of the administered doses (Fig. 8a). Liver function markers, including AST and ALT, were assessed on days 8 and 35 following dosing on days 1 and 7. Both the 15 μg and 50 μg dose groups displayed AST and ALT levels comparable to those in the vehicle group, with all values remaining within the normal range (Fig. 8b and c). These results highlight the favorable safety profile of CpG7909 lipoplex administered intravenously.

## Discussion

In this study, we developed a cationic lipid-based CpG7909 lipoplex formulation that demonstrated enhanced cellular uptake and TLR9 activation in vitro. Systemic administration of this formulation enabled preferential accumulation in both tumor tissue and key immune organs, including the spleen, and was associated with increased CD8 + T-cell infiltration into the TME, indicative of a potential transition from cold to hot tumor phenotypes. Biodistribution data confirmed this pattern of preferential accumulation, likely resulting from passive processes such as the enhanced permeability and retention (EPR) effect rather than active targeting. This approach demonstrated notable therapeutic efficacy in preclinical models against tumors resistant to anti–PD-1 antibodies and those with deep metastases. Previous studies have shown that liposomal CpG ODN formulations can enhance antitumor activity following subcutaneous (SC) or intratumoral (IT) administration^40,52^. Our findings extend this concept by developing an intravenous CpG7909 lipoplex that promotes CD8⁺ T-cell infiltration into the tumor microenvironment and demonstrates synergy with anti–PD-1 therapy in resistant, deep-seated, and metastatic models. This comparison provides contextual support for our work and highlights the novel aspects of systemic CpG7909 lipoplex delivery.

In our study, HEK293 TLR9 reporter assays demonstrated that the CpG7909 lipoplex induced stronger NF-κB activation than free CpG7909, providing initial evidence of enhanced potency. We employed a HEK293-derived TLR9 reporter line in which NF-κB-driven luciferase serves as a downstream read-out of TLR9 activation. Such systems (e.g., HEK-Blue™ hTLR9, TLR9/NF-κB Reporter HEK293) are widely used in the field for screening CpG ODN agonists, as the engineered expression of TLR9 allows specific stimulation to be coupled to NF-κB reporter output. Because NF-κB is a shared downstream node for multiple pathways, this approach provides an indirect measure of TLR9 activation; complementary assays such as assessing MyD88 or IRAK phosphorylation, or using TLR9 inhibitors, would be valuable to confirm signal specificity. Future work will therefore incorporate these complementary assays to verify TLR9-specific signaling and to strengthen mechanistic understanding of CpG7909 lipoplex–mediated immune activation.

To further validate these findings in a more physiologically relevant context, uptake studies were also conducted in RAW-Blue™ macrophages, which endogenously express TLR9 and exhibit phagocytic activity, thereby supporting the physiological relevance of these findings beyond engineered reporter systems. In addition, supplementary data from one experiment (Supplementary Fig. S1) show that mouse ODN1826 (murine CpG7 ODN) lipoplex induced higher TNF-α secretion than free ODN1826 in DC2.4 dendritic cells (*n* = 1), thus indicating that the lipoplex formulation can enhance immune activation in physiologically relevant immune cells. Although this experiment was performed once and is therefore presented as supportive data, it further indicates that the lipoplex formulation can enhance immune activation in physiologically relevant immune cells. Nevertheless, broader validation in other primary immune subsets, such as dendritic cells and B cells, will be necessary in future studies to strengthen mechanistic insights and translational applicability.

In summary, the reduced EC_50_ of CpG7909 lipoplex compared with free CpG7909 may be partly attributable to enhanced intracellular uptake, as suggested by increased fluorescence signals in RAW-Blue™ macrophages. However, prior studies have also demonstrated that CpG presented in lipoplex or spherical nucleic acid formats can enhance TLR9 activation beyond uptake alone, suggesting that both uptake and structural presentation contribute to the observed activity^53^.

Beyond these cellular assays, TLR9 agonists not only promote the production of cytokines and proinflammatory chemokines but also significantly enhance the phagocytosis and activation of antigen-presenting cells^47,54,55^. TLR9 activation enhances recruitment of macrophages and/or neutrophils, thereby improving antigen uptake, processing, and presentation^56^. This mechanism strengthens communication between immune cells, thereby increasing the efficiency and intensity of the immune response^57^. In particular, stimulation of human PBMCs with the CpG7909 lipoplex resulted in greater production of cytokines and proinflammatory chemokines, especially MIP-1α and MIP-1β, than that observed with free CpG7909. These chemokines are critical for leukocyte chemotaxis and immune cell activation, facilitating the recruitment and activation of macrophages and/or neutrophils and enhancing antigen presentation and CD8^+^ T-cell-mediated adaptive immune responses^58^. In the PBMC assays, CpG7909 lipoplex induced broad cytokine responses, reflecting activation of multiple immune cell types. Because PBMCs are heterogeneous, our data could not determine which subsets (e.g., T cells, B cells, monocytes, NK cells) were the predominant contributors. Previous studies have demonstrated that distinct PBMC subpopulations respond differently to CpG stimulation, with B cells and plasmacytoid dendritic cells (pDCs) serving as the primary mediators of TLR9-dependent cytokine production and antigen presentation, while monocytes and NK cells contribute to secondary activation through cytokine cross-talk^59,60^. In this study, PBMCs were intentionally used as a mixed population to capture the integrated immune response that would occur in vivo and to better reflect the physiological context expected in human subjects. Future studies employing single-cell or multiplexed analytical approaches will be necessary to delineate the cell-type–specific mechanisms underlying CpG7909 lipoplex activity.

TILs migrate through the bloodstream into the TME, where they recognize and eliminate cancer cells, which is a crucial step in anticancer immunity^61–63^. Clinical data indicate that higher TIL counts are correlated with improved treatment outcomes and prognosis^61,62,64^. For example, colorectal cancer patients with MSI-H exhibit better prognoses, as immunohistochemical (IHC) analyses have shown that MSI-H tumors contain more infiltrating lymphocytes than microsatellite stable (MSS) tumors. These findings confirm that MSI-H tumors are more immunogenic and more responsive to ICIs^65–67^. The pivotal role of TILs in antitumor activity was further validated in March 2024, when the FDA approved the first TIL-based immunotherapy product, lifileucel (Amtagvi), for cancer treatment. Clinical trial results revealed that among 73 melanoma patients with recurrent disease treated with lifileucel, nearly one-third experienced tumor shrinkage, with a subset achieving complete tumor regression^68^. Additionally, approximately 40% of patients showed no disease progression within a year of receiving lifileucel, demonstrating that increased TILs within tumors significantly contribute to tumor reduction and the prevention of disease progression.

Notably, co-administration of CpG7909 lipoplex with anti-PD1 antibodies significantly enhanced the CR rate in a CT26 tumor model that exhibited resistance to PD-1 blockade alone. The combination treatment increased CR rates from 0 to 75% compared with anti-PD1 monotherapy, and from 12.5 to 75% when compared with free CpG7909 combined with anti-PD1. This enhanced antitumor effect correlated with a marked increase in CD8^+^ T-cell infiltration into the TME. Tumors in the combination group contained over twice as many CD8^+^ T cells as those in the other groups. These findings suggest that the use of CpG7909 lipoplex can increase the therapeutic efficacy of anti-PD1 antibodies by increasing the number of TILs in the TME, thereby addressing the challenge of “cold” tumors that are typically unresponsive to current anti-PD1 therapies. In the same tumor model and under identical treatment conditions, IFN-γ ELISPOT assays on splenocytes (Fig. 5c) demonstrated robust antigen-specific T-cell responses largely mediated by CD8⁺ effector cells, suggesting that infiltrating CD8⁺ T cells were functional and contributed to tumor regression. Furthermore, higher intratumoral CD8⁺ TIL levels are widely recognized as a surrogate marker of antitumor immunity and predict improved responses and clinical outcomes with immune checkpoint inhibitors^69,70^. We acknowledge, however, that enumeration of intratumoral CD8⁺ T cells (Fig. 4a) alone does not confirm their functional status. Because ELISPOT primarily reflects systemic rather than intratumoral responses, future studies will include direct functional profiling of tumor-infiltrating CD8⁺ T cells (e.g., activation markers, cytokine secretion, and cytotoxicity assays) to confirm their local activity.

Our findings suggest that CpG7909 lipoplex enhances the efficacy of anti–PD-1 therapy by priming innate immunity, promoting cytokine production, and increasing CD8⁺ T-cell infiltration, which collectively support more effective T-cell–mediated antitumor responses. Mechanistically, CpG7909 lipoplex converts immunologically “cold” tumors into a more “hot,” T-cell–inflamed phenotype, thereby creating a microenvironment more responsive to immune checkpoint blockade. In this context, CpG7909 amplifies antigen presentation and T-cell priming, while PD-1 blockade restores the effector function of exhausted T cells. This complementary mechanism likely underlies the observed synergy between CpG7909 lipoplex and PD-1 inhibition. Nevertheless, the detailed molecular and cellular interactions remain to be fully elucidated, and future studies will incorporate comprehensive immune profiling and functional analyses to further define the pathways responsible for the enhanced efficacy of the combination therapy.

Vaccination-based strategies are known to activate dendritic cells and induce robust CD4 + and CD8 + T-cell responses, promote infiltration into the TME, and maintain immune responses, ultimately generating effective antitumor immunity and immune memory. TLR9 agonists are employed as adjuvants in tumor vaccines, and studies have shown that TLR9 agonists can induce antigen-specific memory CD8^+^ T cells through dendritic cell activation. In our study, mice that achieved complete tumor remission following anti-PD1 and CpG7909 lipoplex treatment showed no tumor recurrence upon CT26 rechallenge at day 144, well beyond the typical 60–120-day observation period in most murine tumor models. This finding suggests the establishment of long-term immune protection, consistent with previous studies indicating that durable tumor control and resistance to rechallenge are accepted indicators of persistent T-cell–mediated memory^71,72^. Furthermore, splenocytes from cured mice secreted IFN-γ in response to AH1, a CT26-derived antigen, confirming the induction of functional, antigen-specific immune responses. Together, these results support the notion that the observed long-term antitumor immunity arises from sustained, antigen-specific T-cell activation induced by CpG7909 lipoplex in combination with immune checkpoint blockade. Nevertheless, confirming whether such immune memory can persist over months or years in patients will require long-term studies across multiple tumor models and clinical validation.

While intratumoral injection of TLR9 agonists (such as MGN1703 and IMO-2125) has shown preliminary clinical efficacy in treating superficial tumors, repeated administration remains clinically challenging, particularly for deep-seated or metastatic tumors. In the CT26 lung metastasis model, combination therapy with intravenous CpG7909 lipoplex and anti-PD1 resulted in significantly reduced tumor bioluminescence signals and a 90% CR rate, which wasmore than double the CR rate achieved with chemotherapy or anti-PD1 monotherapy.

These results demonstrate potent and systemic antitumor activity in metastatic settings. The intravenous CpG7909 lipoplex enables efficient delivery and preferential distribution to tumors and immune organs such as the spleen, which may underlie the observed therapeutic benefit in deeply metastatic and PD-1–resistant tumors. The high complete response (CR) rate achieved in the CT26 lung metastasis model provides compelling preclinical evidence of translational potential, consistent with clinical observations that CR serves as a strong prognostic marker in immunotherapy trials such as KEYNOTE-001 and CheckMate 067. While results from murine models cannot be directly extrapolated to human outcomes, these findings support the potential of CpG7909 lipoplex to enhance systemic antitumor immunity. Future studies with extended follow-up in metastatic models will be necessary to confirm the durability of these responses. Although immune profiling was not performed in the lung metastasis model, our subcutaneous CT26 study showed that intravenous administration of CpG7909 lipoplex enhanced intratumoral CD8⁺ T-cell infiltration and elicited systemic IFN-γ responses in the spleen. These findings highlight the systemic immunostimulatory potential of CpG7909 lipoplex, and future work will include detailed immune profiling in metastatic settings to further clarify its mechanism of action.

The pharmacokinetic and biodistribution characteristics of CpG7909 lipoplex further support its suitability for systemic delivery. Compared with free CpG7909, the lipoplex exhibited prolonged circulation and sustained tissue retention, consistent with the behavior of lipid-based formulations. Enhanced accumulation was observed in immune-relevant organs such as the spleen and lymph nodes, as well as within tumor tissue, and these differences persisted across all examined time points from 4 to 72 h. This prolonged and preferential distribution suggests that the lipoplex remains structurally relatively stable during circulation, enabling effective delivery to immune organs and tumor sites, thereby facilitating systemic immune activation. However, the precise proportion of intact versus dissociated CpG7909 in vivo remains undefined. Future work will include direct quantification of encapsulated CpG7909 in plasma and tissues to confirm the stability and integrity of the lipoplex formulation.

To further evaluate its translational potential,, safety assessments of CpG7909 lipoplex administered via intravenous injection further supported its potential for clinical applications. BALB/c mice receiving two consecutive weekly doses of CpG7909 lipoplex (15 or 50 μg/mouse) showed no noticeable or apparent body weight changes, and liver function markers (AST and ALT) remained within the normal range throughout the study. Given that biodistribution analysis revealed high accumulation of CpG7909 lipoplex in the liver, this organ was a particular focus of our preliminary safety evaluation.

In addition, common adverse effects previously reported for free CpG7909, such as splenomegaly and extramedullary hematopoiesis^73^, were not observed in CpG7909 lipoplex–treated mice (Supplementary Fig. S2). Spleen weight showed no significant difference compared with the vehicle group, and histopathological examination of spleen tissue (H&E staining, 400 ×) revealed no abnormal hematopoietic activity or tissue enlargement.

While no adverse findings were observed in this preliminary safety assessment, we recognize that these limited evaluations are insufficient to fully establish the overall safety profile of the formulation. Comprehensive toxicology studies, including liver histopathology, full serum chemistry, and hematology, will therefore be essential in future development.

Together, these findings demonstrate that intravenous delivery of CpG7909 lipoplex mitigates the systemic toxicities commonly associated with TLR9 agonists while maintaining potent immune activation, thereby confirming its safety, tolerability, and ability to overcome the major limitations of intratumoral administration for metastatic tumors.

## Conclusions

The CpG7909 lipoplex represents a promising platform for systemic delivery of TLR9 agonists, with the potential to enhance the therapeutic efficacy of immune checkpoint blockade in tumors that are otherwise unresponsive to current immunotherapies. Its capacity to convert cold tumors into an immunologically active state underscores its clinical relevance, particularly in the context of deep-seated or metastatic cancers. Further preclinical and clinical studies focused on safety profiling, immune modulation, and dosing optimization will be essential to advance this approach toward clinical application.

## Supplementary Information

Below is the link to the electronic supplementary material.

Supplementary Material 1

Supplementary Material 2

Supplementary Material 3

## Data Availability

Data is provided within the manuscript or supplementary information files.

## Abbreviations

AH1: Immunodominant peptide from gp70 envelope protein of CT26 tumor cells
ALT: Alanine aminotransferase
APC: Antigen-presenting cell
AST: Aspartate aminotransferase
AST-008: Advanced Spherical TLR9 agonist-008 (a nanoparticle-based TLR9 agonist)
ATCC: American Type Culture Collection
AUC: Area under the curve
BALB: BALB/c (Bagg Albino) mouse strain
CD: Cluster of differentiation
CR: Complete response
CT26: Colon carcinoma cell line (murine)
DOTAP: 1,2-Dioleoyl-3-trimethylammonium-propane
DSPE-PEG: 1,2-Distearoyl-sn-glycero-3-phosphoethanolamine-polyethylene glycol
EE: Encapsulation efficiency
ELISPOT: Enzyme-linked immunospot assay
EMT6: Murine breast cancer cell line
F-CpG7909: Free CpG7909 (unformulated)
FDA: U.S. Food and Drug Administration
H&E: Hematoxylin and eosin
HEK293: Human embryonic kidney 293 cells
HEPES: 4-(2-Hydroxyethyl)-1-piperazineethanesulfonic acid
ICIs: Immune checkpoint inhibitors
IFN-γ: Interferon gamma
IHC: Immunohistochemistry
IL: Interleukin
IVIS: In vivo imaging system
LAG-3: Lymphocyte-activation gene 3
MIP-1α/β: Macrophage inflammatory protein 1 alpha/beta
MRT: Mean residence time
MSI-H: Microsatellite instability-high
MSS: Microsatellite stable
N/P ratio: Nitrogen/phosphate ratio
NF-κB: Nuclear factor kappa-light-chain-enhancer of activated B cells
NK: Natural killer (cell)
PBMC: Peripheral blood mononuclear cell
PBS: Phosphate-buffered saline
PD-1: Programmed death-1
PD-L1: Programmed death-ligand 1
PDI: Polydispersity index
PEG: Polyethylene glycol
PMA: Phorbol 12-myristate 13-acetate
SD: Standard deviation
SEM: Standard error of the mean
SPE: Solid-phase extraction
TGI: Tumor growth inhibition
TILs: Tumor-infiltrating lymphocytes
TLR9: Toll-like receptor 9
TME: Tumor microenvironment
Th1: T helper 1
ip: Intraperitoneal
iv: Intravenous
