# Supplementary material for: Intravenous administration of CpG7909 lipoplex enhances anti-PD1 immunotherapy by modulating the tumor microenvironment and inducing durable tumor regression
Source: Sci Rep. 2025 Nov 25;15:45354. doi: 10.1038/s41598-025-29622-x (PMC12749086; doi:10.1038/s41598-025-29622-x)
Supplement: Supplementary file 1 — Supplementary Material 1 [file 41598_2025_29622_MOESM1_ESM.docx]

Method: The immortalized DC2.4 mouse dendritic cell line (SCC142) was obtained from Merck and cultured in RPMI-1640 medium (Corning) supplemented with 10% (v/v) fetal bovine serum (FBS; Thermo Fisher Scientific), 2.5% (v/v) HEPES buffer (1 M; Corning), 1% (v/v) MEM Non-Essential Amino Acids Solution (100×; Corning), 1% (v/v) Penicillin-Streptomycin Solution (100×; Corning), and 0.00054% (v/v) 2-mercaptoethanol (Thermo Fisher Scientific).A total of 1.5 × 10⁵ DC2.4 cells were seeded in 24-well plates and incubated overnight prior to treatment. At different time points, the treatment medium was replaced with fresh medium without OND, and the culture supernatant was collected for subsequent analysis. Mouse TNF-α levels were determined using a Mouse TNF Alpha Uncoated ELISA Kit (Thermo

Fisher Scientific) according to the manufacturer’s protocol.

Based on the TNF-α levels observed in ODN-stimulated DC2.4 cells, ODN 1826 SNA appears to undergo earlier cellular internalization than free ODN 1826, resulting in enhanced TNF-α secretion.


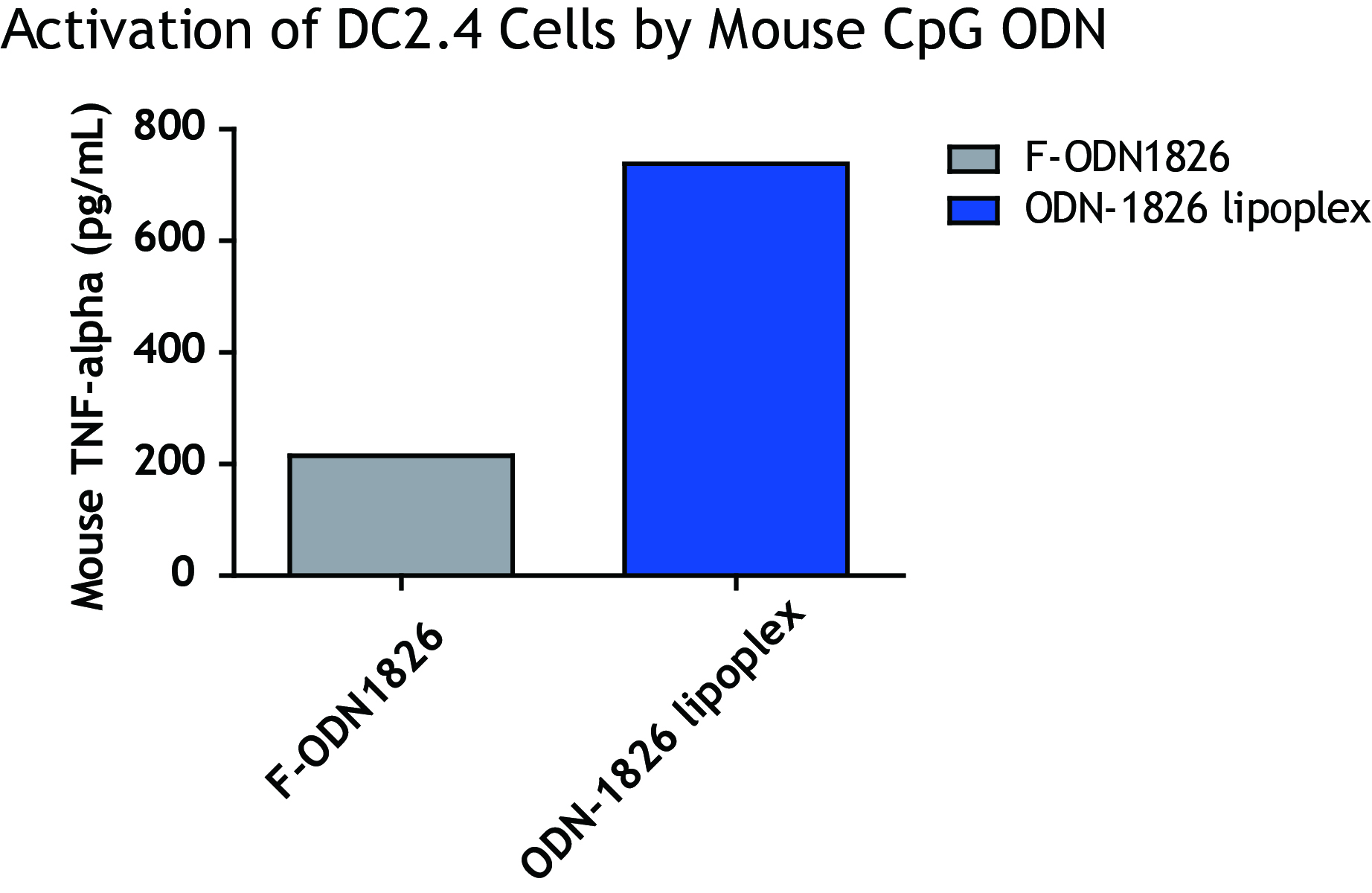


Supplementary Fig. S1. Activation of DC2.4 cells by mouse ODN1826 lipoplex.

Mouse DC2.4 cells were treated with free ODN1826 (F-ODN1826) or ODN1826 lipoplex, and TNF-α secretion was quantified by ELISA. The ODN1826 lipoplex induced a higher level of TNF-α production than free ODN1826, indicating enhanced activation of dendritic cells. Data represent a single experiment (n = 1); therefore, these results are presented as supportive findings to illustrate the trend of enhanced immune activation in a physiologically relevant murine immune cell line.
