# Supplementary material for: Intravenous administration of CpG7909 lipoplex enhances anti-PD1 immunotherapy by modulating the tumor microenvironment and inducing durable tumor regression
Source: Sci Rep. 2025 Nov 25;15:45354. doi: 10.1038/s41598-025-29622-x (PMC12749086; doi:10.1038/s41598-025-29622-x)
Supplement: Supplementary file 2 — Supplementary Material 2 [file 41598_2025_29622_MOESM2_ESM.docx]

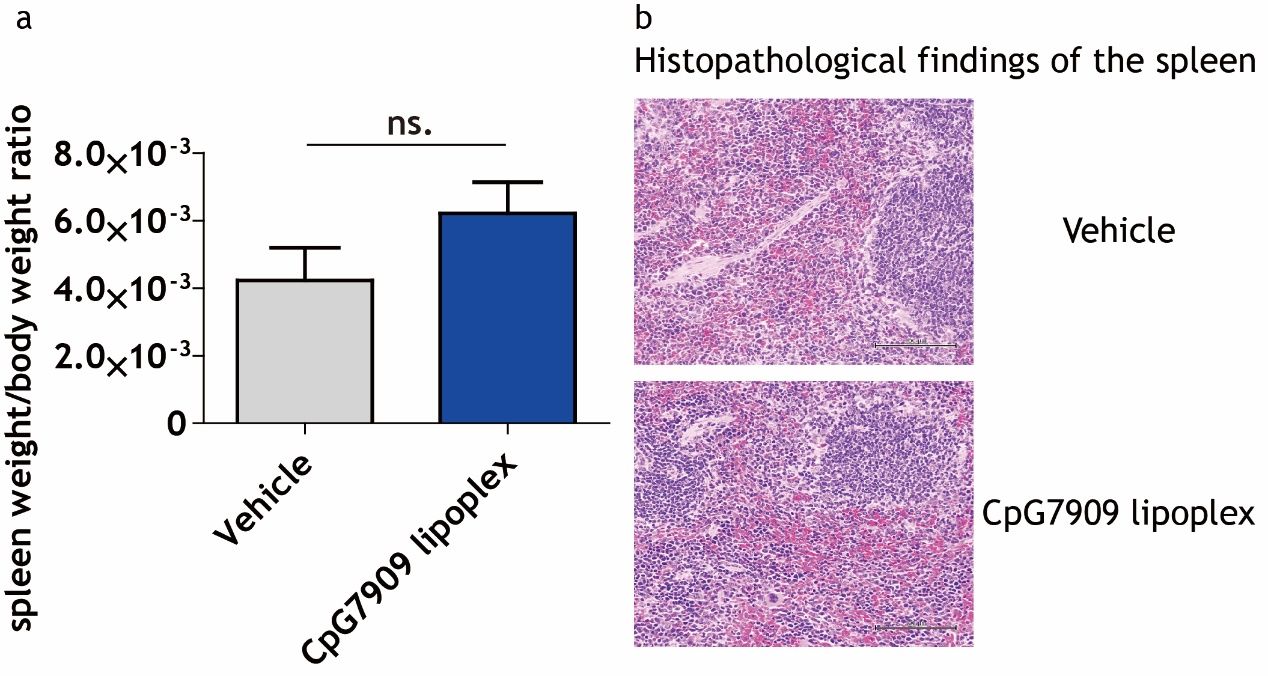


Supplementary Fig. S2. Spleen evaluation following intravenous administration of CpG7909 lipoplex.

(a) Spleen weight–to–body weight ratio of BALB/c mice after two consecutive weekly doses of CpG7909 lipoplex (15 μg/mouse, intravenous). No significant difference was observed compared with the vehicle group (ns., not significant; unpaired two-tailed t-test).(b) Representative histopathological images of spleen tissue (H&E staining, 400×). Normal splenic architecture was observed in both groups without evidence of splenomegaly or abnormal hematopoietic activity. Scale bars = 100 μm.
