# Supplementary material for: Intravenous administration of CpG7909 lipoplex enhances anti-PD1 immunotherapy by modulating the tumor microenvironment and inducing durable tumor regression
Source: Sci Rep. 2025 Nov 25;15:45354. doi: 10.1038/s41598-025-29622-x (PMC12749086; doi:10.1038/s41598-025-29622-x)
Supplement: Supplementary file 3 — Supplementary Material 3 [file 41598_2025_29622_MOESM3_ESM.docx]

**Supplementary Table S1 Summary of Pharmacokinetic Parameters**

| **CT26 tumor mice** | **F-CpG7909** | **CpG7909 Lipoplex** |
| --- | --- | --- |
| T_1/2_（hr） | 13.3±1.0 | 16.0±4.4 |
| C_0_（ng/mL） | 1479.2±194.4 | 1819.2±432.3 |
| AUC_all_（hr*ng/mL） | 5264.5±3089.5 | 15318.6±7015.7 |
| V_ss_（mL） | 44.0±15.1 | 20.1±3.2 |
| Cl（mL/hr） | 3.4±1.4 | 1.1±0.5 |
| MRT_last_（hr） | 11.3±1.4 | 17.5±6.5 |
